# Paired pulse transcranial magnetic stimulation in the assessment of biceps voluntary activation in individuals with tetraplegia

**DOI:** 10.3389/fnhum.2022.976014

**Published:** 2022-11-03

**Authors:** Thibault Roumengous, Bhushan Thakkar, Carrie L. Peterson

**Affiliations:** ^1^Department of Biomedical Engineering, Virginia Commonwealth University, Richmond, VA, United States; ^2^Department of Physical Therapy, Virginia Commonwealth University, Richmond, VA, United States

**Keywords:** central activation ratio, spinal cord injury, interpolated twitch technique, quadriplegia, voluntary contraction, motor evoked potential

## Abstract

After spinal cord injury (SCI), motoneuron death occurs at and around the level of injury which induces changes in function and organization throughout the nervous system, including cortical changes. Muscle affected by SCI may consist of both innervated (accessible to voluntary drive) and denervated (inaccessible to voluntary drive) muscle fibers. Voluntary activation measured with transcranial magnetic stimulation (VA_TMS_) can quantify voluntary cortical/subcortical drive to muscle but is limited by technical challenges including suboptimal stimulation of target muscle relative to its antagonist. The motor evoked potential (MEP) in the biceps compared to the triceps (i.e., MEP ratio) may be a key parameter in the measurement of biceps VA_TMS_ after SCI. We used paired pulse TMS, which can inhibit or facilitate MEPs, to determine whether the MEP ratio affects VA_TMS_ in individuals with tetraplegia. Ten individuals with tetraplegia following cervical SCI and ten non-impaired individuals completed single pulse and paired pulse VA_TMS_ protocols. Paired pulse stimulation was delivered at 1.5, 10, and 30 ms inter-stimulus intervals (ISI). In both the SCI and non-impaired groups, the main effect of the stimulation pulse (paired pulse compared to single pulse) on VA_TMS_ was not significant in the linear mixed-effects models. In both groups for the stimulation parameters we tested, the MEP ratio was not modulated across all effort levels and did not affect VA_TMS_. Linearity of the voluntary moment and superimposed twitch moment relation was lower in SCI participants compared to non-impaired. Poor linearity in the SCI group limits interpretation of VA_TMS_. Future work is needed to address methodological issues that limit clinical application of VA_TMS_.

## Introduction

### Utility of cortical and peripheral voluntary activation after spinal cord injury

Voluntary activation (VA) quantifies the level of voluntary neural drive to muscle during maximum voluntary effort (Gandevia et al., 1995). A deficit in voluntary activation is indicated when muscle force during maximum voluntary effort is further increased by artificial stimulation (Herbert and Gandevia, 1999). A deficit in VA may result if there exists denervated muscle fibers inaccessible to voluntary drive, or suboptimal motor unit firing; both denervation and suboptimal firing affect muscle after spinal cord injury (SCI) (Berman et al., 1996). Thus, non-invasive measures of VA are useful in guiding SCI rehabilitation (Kim et al., 2015; Peterson et al., 2017). VA of a specific muscle or muscle group can be measured via peripheral nerve stimulation (VA_PNS_) superimposed on an isometric maximum voluntary contraction (MVC) (Allen et al., 1995; Vøllestad, 1997). However, VA_PNS_ is limited in its ability to reveal cortical (i.e., originating from the motor cortex) deficits in VA, versus spinal and peripheral factors (i.e., function of spinal motoneurons and neuromuscular junction) (Allen et al., 1995; Hunter et al., 2006). After SCI, motoneuron death occurs at and around the level of injury (Lin et al., 2007; Grumbles and Thomas, 2017), which ultimately induces neuroplasticity throughout the nervous system, including cortical plasticity (Nardone et al., 2013). One way to assess cortical plasticity that may affect VA is with transcranial magnetic stimulation (TMS) of the motor cortex superimposed on voluntary contraction (Kobayashi and Pascual-Leone, 2003; Todd et al., 2003; Hallett, 2007). VA assessed with TMS (VA_TMS_) can elucidate the site of impairment in voluntary drive (Todd et al., 2004). Together, both VA_TMS_ and VA_PNS_ may provide a more comprehensive indicator of VA after SCI.

### Challenge in measuring VA_TMS_ after spinal cord injury

While VA_PNS_ methodology can assess muscle affected by SCI (Kim et al., 2015; Peterson et al., 2017; Jakubowski et al., 2018), technical challenges in measuring VA_TMS_ exist after SCI (Thomas et al., 1997) and in the assessment of other patient populations (Todd et al., 2003, 2016; Hunter et al., 2006; Smith et al., 2007; Kotan et al., 2015; Cadigan et al., 2017; Mira et al., 2017; Ansdell et al., 2019). One important limitation of VA_TMS_ is the recruitment of muscles other than the target muscle because TMS over the motor cortex can stimulate neighboring cortical neural pathways projecting to agonistic and antagonistic muscles (Todd et al., 2006). This lack of precision is due, in part, to the high stimulation intensities needed to evoke measurable force responses, especially in patients with neurologic impairment (Thomas et al., 1997; Todd et al., 2016; Cadigan et al., 2017). Greater stimulus intensities are associated with greater stimulus spread in the brain (Brasil-Neto et al., 1992). The motor evoked potential (MEP) in response to TMS of the target muscle compared to its antagonist’s MEP can indicate, to some degree, the focality of stimulation and excitability of pathways projecting to the target muscle relative to its antagonists (Nielsen and Kagamihara, 1993; Hansen et al., 2002; Todd et al., 2016; Kesar et al., 2019). Isolated recruitment of the target muscle with TMS, while ideal, is difficult with currently available TMS devices (Deng, 2013; Rotenberg et al., 2014; Todd et al., 2016). As such, Todd et al. (2016) suggest a realistic compromise of isolated recruitment of the target muscle when its MEP amplitude reaches ≥50% of the muscle’s maximal compound motor action potential (Mmax) and the antagonist MEP amplitude is ≤20% of Mmax (Todd et al., 2016). This compromise can be achieved in non-impaired muscle by adjusting TMS intensity (Todd et al., 2006, 2016; Kotan et al., 2015). However, this is more difficult in the assessment of muscle affected by SCI (Thomas et al., 1997; Oudega et al., 2012; Angeli et al., 2014; Nardone et al., 2015). For example, in assessing the severely paralyzed triceps in individuals with C7 or higher tetraplegia, the TMS intensity could not be adjusted to elicit appropriate responses in the triceps to estimate VA_TMS_ (Thomas et al., 1997). Thus, additional considerations and modifications to existing VA_TMS_ protocols are needed to assess VA_TMS_ in muscle affected by SCI. As a first step, we focus on improving the methodology to assess VA_TMS_ of the biceps brachii in individuals with C5 and C6 tetraplegia because: a) the biceps is innervated at the C5 and C6 levels such that some biceps function typically remains (Ditunno et al., 1992; Calancie et al., 2004), which may increase the feasibility of assessing VA_TMS_ after SCI, b) the biceps is important for upper limb function (Crago et al., 1998), and c) the antagonist triceps is typically more severely affected by SCI (Sangari and Perez, 2020), which may also increase the feasibility of assessing VA_TMS_ after SCI.

### Proposed approach to isolate recruitment of the biceps in measuring VA_TMS_ after spinal cord injury

One approach to better isolate TMS recruitment of the biceps to assess biceps VA_TMS_ after SCI is to increase the motor response to TMS in the biceps relative to the triceps; the degree to which that is achieved can be measured by the ratio of the biceps MEP relative to the triceps MEP (i.e., biceps MEP amplitude divided by triceps MEP amplitude). Paired pulse TMS techniques, which can modulate MEP amplitudes, can potentially increase the biceps/triceps MEP ratio relative to single pulse TMS in the assessment of VA_TMS_ after SCI. Paired pulse TMS techniques consist of a conditioning stimulus followed by a test stimulus with a specific inter-stimulus interval (ISI) between the two stimuli (Kujirai et al., 1993). At ISIs ranging from 10 to 30 ms, MEPs are typically increased in the resting, non-impaired muscle relative to single pulse TMS through the physiologic mechanism referred to as intracortical facilitation (ICF) (Nakamura et al., 1997). At shorter ISIs (ranging from 1 to 5 ms), MEPs are decreased in the resting muscle relative to single pulse TMS through the physiologic mechanism referred to as short intracortical inhibition (SICI) (Nakamura et al., 1997; Ziemann, 1999). Paired pulse TMS techniques have been applied to non-impaired muscle during low levels of voluntary contraction (e.g., contraction ≤ 25% MVC, Hunter et al., 2016; Temesi et al., 2017), and sparsely applied to muscle affected by tetraplegia at rest (Cirillo et al., 2016). While MEP amplitudes in non-impaired muscle become saturated at increasing levels of voluntary contraction (e.g., Valls-Sole et al., 1994; Van den Bos et al., 2018), muscle affected by SCI may not demonstrate MEP saturation given the corticospinal reorganization that occurs (Oudega et al., 2012; Cirillo et al., 2016). Currently, the effect of paired pulse TMS on biceps and triceps MEPs at high levels of voluntary contraction is unknown in individuals with tetraplegia; this is relevant because VA_TMS_ is assessed by superimposing TMS on voluntary effort levels ranging from 50 to 100% MVC to extrapolate the relationship between the voluntary moment and superimposed twitch (SIT) moment (Todd et al., 2003, 2004, 2016; Cadigan et al., 2017). Further, it is unknown whether an increased biceps/triceps MEP ratio across effort levels can improve the estimation of VA_TMS_ after SCI.

### Purpose and hypotheses

The purpose of this preliminary study was to determine the relationship between biceps VA_TMS_ and the biceps/triceps MEP ratio in individuals with C5 and C6 tetraplegia. Although we focus on the relationship between biceps VA_TMS_ and the biceps/triceps MEP ratio in the current study, we do so with the intent that our approach may be transferable to evaluate VA_TMS_ in other muscles affected by SCI. Paired pulse TMS was tested as a method to modulate the biceps/triceps MEP ratio across effort levels of 50, 75, and 100% MVC needed to assess VA_TMS_. In evaluating individuals with C5 and C6 tetraplegia, we hypothesized that paired pulse TMS with ISI that can facilitate biceps MEPs would increase the biceps/triceps MEP ratio across all effort levels relative to single pulse TMS biceps. Also, we hypothesized that paired pulse TMS with ISI that can inhibit biceps MEPs would decrease the biceps/triceps MEP ratio across effort levels relative to single pulse TMS in individuals with tetraplegia. Finally, we hypothesized that the biceps/triceps MEP ratio would affect VA_TMS_ in individuals with tetraplegia. The rationale behind this hypothesis is that the biceps/triceps MEP ratio may indicate the amount of cortical stimulation to the biceps relative to the triceps, with greater biceps cortical stimulation affecting biceps VA_TMS_.

## Materials and methods

### Experiment overview

**FIGURE 1:**
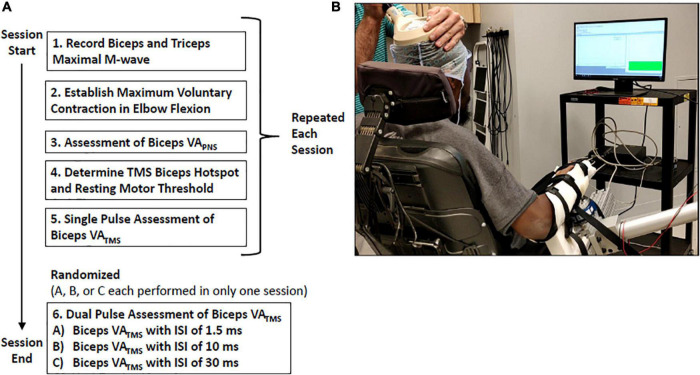
**(A)** Experimental protocol diagram representing the data collected during a single session. Participants completed two or three sessions in total. **(B)** Experimental setup: Participants received visual feedback of their voluntary elbow flexion moment as a thermometer-like gauge.

In each session, participants completed trials to assess VA_TMS_, and also VA_PNS_ to provide context for the VA_TMS_ results. Elbow joint force and moment data, and elbow flexor (biceps brachii) and extensor (triceps brachii) electromyographic (EMG) data were collected from ten individuals with C5 or C6 SCI (Table 1). Inclusion criteria required SCI participants to be between the ages of 18 and 65 years old with a low cervical spinal injury at levels C5–C6 as indicated by the International Standards for Neurological Classification of Spinal Cord Injury (ISNCSCI), and at least 1-year post-injury. Exclusion criteria included metal implants in the head and the inability to generate a visible contraction of the biceps. Data from one participant with SCI (#10) was excluded from the data analyses because TMS was unable to elicit measurable moment twitches from the elbow flexors. Ten non-impaired individuals (four females, six males, average age 22.7 ± 2.5 years) also participated to provide a context for our findings in individuals with SCI. SCI participants completed either two or three sessions; all non-impaired participants completed three sessions (Table 1). During each session, participants were seated in a chair with their dominant arm supported against gravity in an isometric posture, the elbow flexed at 90 degrees, and the forearm supinated (Figure 1). Participants’ biceps and triceps maximal M-wave responses, elbow flexion MVC, VA_PNS_, and VA_TMS_ were measured during each of the three sessions; only one of the three paired pulse VA_TMS_ protocols were assessed per session to limit fatigue and the number of stimulus events (Figure 1). Each session took approximately 3 h. This study was approved by the Virginia Commonwealth University Institutional Review Board (HM20010929). Written consent was obtained from all participants.

**TABLE 1:** Ten individuals with tetraplegia following cervical spinal cord injury (SCI) were recruited to participate in the study.

Participant #	Sex	Age	Injury level	ISNSCI	Years since SCI	Cause of SCI	MVC (Nm)	Medications
1	F	52	C6	A	15	MVA	47.6 ± 1.89	BAC
2	F	53	C6	D	7	Spinal stenosis	57.2 ± 2.40	BAC
3	M	42	C5	A	12	MVA	35.0 ± 1.93	BAC
4	M	45	C6	D	5	Transverse myelitis	52.4 ± 2.69	None
5	F	54	C6	A	13	MVA	20.6 ± 2.27	BAC
6*	M	34	C5	A	16	MVA	21.0 ± 1.59	BAC, OX
7*	M	26	C6	A	6	Fall	80.7 ± 2.79	None
8*	M	33	C5	D	3	MVA	93.6 ± 2.84	None
9	M	32	C5	B	9	Fall	40.5 ± 1.68	None
10**	M	28	C5	B	4	MVA	2.0 ± 0.07	BAC

Maximum voluntary elbow moments presented here were measured at 90° of elbow flexion.

SCI: spinal cord injury; MVC: maximal voluntary contraction; ISNCSCI: International standards for neurological classification of spinal cord injury; MVA: motor vehicle accident; BAC: Baclofen, OX: Oxybutynin.

*Only completed two sessions as a result of lab shutdown during COVID-19.

**Excluded data.

### Electromyographic and kinetic recordings

All data were recorded via a custom Spike2 script and a data acquisition system (CED 1401, Cambridge, UK). EMG data were recorded using wireless EMG sensors (Delsys Trigno, Natick, Massachusetts) placed on the participant’s biceps and triceps in a muscle belly tendon arrangement. EMG data were sampled at 2,000 Hz and bandwidth limited to 20–450 Hz. The participant’s forearm was positioned in a custom brace attached to a multi-axis load cell with a measurement range of ± 400 N and digital resolution of 0.1 N (JR3 30E15A4, Woodland, California). A different load cell was used for participants with weaker elbow flexors (JR3 30E12A4, measurement range of ± 100 N and digital resolution of 0.025 N). Three-dimensional force and moment data were recorded at 2,000 Hz and transformed to the elbow joint using standard coordinate transformations to determine the elbow flexor moment (Sciavicco and Siciliano, 2000).

### Compound motor unit action potential recording

Electrical stimuli were delivered using a constant current stimulator (Digitimer DS7AH, Fort Lauderdale, Florida) at 200 V with a 200 μs pulse width. The current delivered ranged from 5 mA (threshold of detection) to 150 mA (procedural maximum). Rectangular 3.3 × 5.3 cm neurostimulation electrodes (Axelgaard 891200, Fallbrook, CA, USA) were placed at Erb’s point (cathode) and the acromion (anode). M-wave recruitment curves were obtained individually for the biceps and then the triceps starting from zero at intervals of 10 mA until a plateau in the M-wave amplitude was reached. Five supramaximal stimuli of 120% of the threshold current were delivered to obtain the maximal M-wave (Mmax) for the biceps and triceps at rest.

### Assessment of VA_PNS_

Participants completed trials during which motor point electrical stimulation was superimposed on isometric MVCs in elbow flexion in order to estimate VA_PNS_. For motor point stimulation, stimulating electrodes were placed over the biceps belly (anode) and distal tendon (cathode). Stimulus intensity was determined by increasing the stimulation current in 10 mA increments until the moment response in the resting biceps reached a plateau. The threshold current (i.e., current corresponding to the start of the moment plateau) was recorded and motor point stimulation intensity was set at 130% of the threshold current (Allen et al., 1998; Hanajima et al., 1998). Stimulation intensity ranged from 80 to 180 mA across both groups. Using visual moment feedback, participants were instructed to perform nine MVCs in elbow flexion during which stimulation was superimposed during and after the voluntary effort. Motor point stimulation with a single pulse (0.2 ms width, DS7AH, Digitimer, UK) was delivered after the participant maintained a voluntary moment ≥95% of their MVC moment for 0.5 s. A second stimulus event (same intensity and pulse width) was delivered 3 s after the first stimulus event while the arm was at rest. Each trial was followed by at least 90 s of rest to mitigate fatigue.

### Transcranial magnetic stimulation

Motor cortex stimulation was delivered using a 126 mm double cone coil and Magstim BiStim^2^ stimulator (Magstim, Whitland, United Kingdom). For all stimuli, a monophasic waveform was applied with the coil held to induce an anterior-to-posterior current across the central sulcus. Motor mapping of the cortical hotspot was performed during each session to obtain the location that evoked the largest peak-to-peak MEP in the biceps relative to the triceps using the lowest stimulation intensity (Oliveri et al., 1999). The hotspot location was then marked on a silicone or plastic cap secured to the participant’s head. Resting motor threshold (RMT) was then determined as the lowest stimulus intensity able to induce MEPs of ≥50 μV in at least 50% of ten stimuli and expressed as a percentage of the maximum stimulator output (%MSO) (Rossini et al., 1999). To reduce the number of stimuli, RMT was identified using maximum likelihood adaptive parameter estimation (Awiszus, 2003).

### Protocol to assess VA_TMS_

Participants started with a quick familiarization phase and warm-up, which consisted of brief submaximal contractions over 2 min. Participants were then instructed to perform three sustained, isometric contractions of the elbow flexors at maximum effort to determine their MVC moment. Contractions were sustained for 3 s while participants received both real-time visual moment feedback, and auditory encouragement (Figure 1B). Real-time visual elbow moment feedback was displayed on a nearby monitor as a thermometer-like bar. The MVC was calculated as the mean elbow flexion moment occurring within a 0.5 s window from the maximal moment value. The average of all three MVC efforts was used in the following VA_TMS_ trials where participants generated a voluntary moment to match a percentage of their MVC moment. Each MVC was separated by 90 s of rest. After locating the cortical hotspot and establishing RMT, VA_TMS_ was assessed. Baseline (single pulse) and modified (paired pulse) VA_TMS_ protocols were assessed in a randomized order, with at least one baseline and one modified protocol per session (Figure 1A). VA_TMS_ protocols consisted of a set of 24 isometric contractions of the elbow flexors during randomized moment-matching trials of 0, 50, 75, or 100% MVC. Trials were separated by at least 90 s of rest to mitigate fatigue. To obtain the SIT moment in the single pulse protocol, a supramaximal (i.e., 120% RMT) TMS pulse was automatically delivered after the participant achieved and maintained ± 2.5% of the target effort for 0.5 s. Paired pulse stimuli were delivered as a conditioning pulse set to 90% RMT followed by a test pulse at 120% RMT. These intensities were based on prior paired pulse TMS studies in healthy muscle at rest (Chen et al., 1998, 2018; Vucic et al., 2009; Kirton and Gilbert, 2016; Opie et al., 2020; Tugin et al., 2021) due to the lack of data describing effects of SICI and ICF protocols on biceps and triceps MEPs in tetraplegia (at rest or during contraction). Conditioning and test stimuli were separated by an ISI of 1.5, 10, or 30 ms. Stimuli with 1–6 ms ISIs can inhibit MEPs while ISIs of 8–30 ms can facilitate MEPs in resting non-impaired muscle (Kujirai et al., 1993; Chen et al., 1998; Hanajima et al., 1998; Ziemann, 1999; Hunter et al., 2016).

### Data and statistical analysis

**FIGURE 2:**
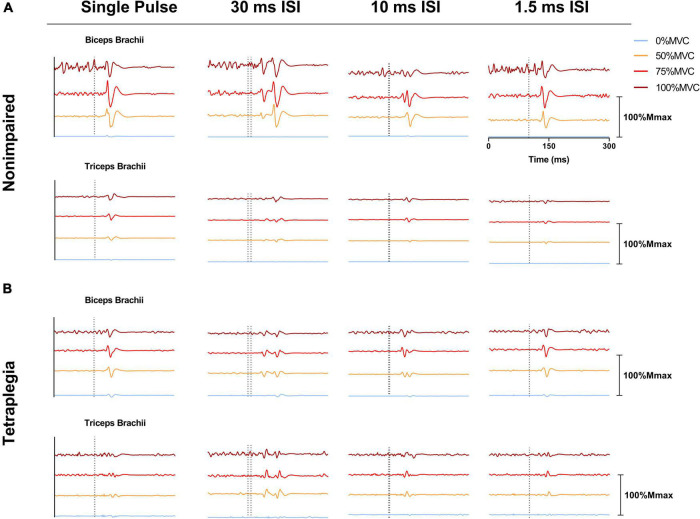
Electromyographic (EMG) traces showing representative MEPs over a 300 ms window across effort levels and stimulation pulses (single pulse 1.5, 10, and 30 ms ISI). **(A)** EMG recordings from the biceps brachii and triceps brachii of a representative non-impaired participant. **(B)** EMG recordings from the biceps brachii and triceps brachii of a representative SCI participant. EMG signals shown were averaged across six trials and normalized to the Mmax of the corresponding session/participant. EMG traces presented within the same subdivision were offset from one another for presentation. The dotted line on one subdivision represents the onset of stimulation.

Force, moment and EMG data were post-processed using a custom MATLAB script. For both single pulse and paired pulse trials, MEPs were determined as the peak-to-peak EMG signal within 100 ms of the cortical stimulation (onset of the first pulse when paired pulse was performed) and subsequently normalized to the Mmax of each session; all MEPs were visually inspected. The MEP ratio for each trial was calculated as the normalized biceps MEP divided by the normalized triceps MEP. EMG traces of representative participants from the non-impaired group and the SCI group are presented in Figure 2. VA_TMS_ was calculated as a percentage using the interpolated twitch technique: VA_TMS_ (%) = (1 – SIT at 100% MVC)/(estimated resting twitch) × 100 (Todd et al., 2003). The resting twitch was estimated using linear regression of the 50–100% MVC efforts (see Todd et al., 2004, method 1 for detailed explanation, and see Supplementary Figures 1–4 (Todd et al., 2004). Finally, the pre-TMS stimulation EMG activity of the biceps and triceps was calculated as the root mean square of the signal during the 50 ms directly before stimulation.

VA_PNS_ superimposed twitch moments were computed for each trial as the difference between the maximum moment occurring within 150 ms after the stimulus event and the pre-stimulus moment. The pre-stimulus moment was computed as the maximal 10 ms moving average moment maintained within 50 ms prior to the stimulus event. The potentiated resting twitch moment was also computed for each motor point stimulation trial. VA_PNS_ was calculated according to Allen et al. (1998)

Linear mixed effects models for each participant group were analyzed to determine the effect of independent variables on VA_TMS_ (the dependent variable). The independent variables were defined as follows: stimulation pulse (single pulse vs paired pulse conditions), block mean biceps/triceps MEP ratio, linearity of the voluntary moment and SIT relation (*R*-value), and RMT. Blocks with low linearity (*r* < 0.8) in the non-impaired group were excluded, similar to previous work assessing non-impaired muscle (Todd et al., 2016); eleven out of 60 blocks were excluded based on low linearity. In the SCI group, analyses to determine relationships with VA_TMS_ included all blocks (i.e., data were not excluded based on linearity). Further, in the linear mixed effects model for the SCI group, linearity (*R*-value) was included as an independent variable to test whether variations in linearity affected the estimation of VA_TMS_. RMTs were added to the models as a continuous covariate to test whether individual responsiveness to TMS (as represented by RMTs) affected VA_TMS_. A random effect was added to account for individual differences that resulted in each participant being assigned a different intercept. *P*-values were obtained via the Kenward-Roger approximation for degrees-of-freedom implemented for linear mixed effect models (Herring, 2013). Comparisons were reported with respect to single pulse VA_TMS_ measures. Two-way ANOVAs with repeated measures were used to compare MEP ratios of single pulse to paired pulse VA_TMS_ conditions (1.5, 10 ms or 30 ms ISI) across effort levels (0, 50, 75, and 100% MVC). Two-way ANOVAs were also used to compare biceps and triceps MEPs between single pulse and paired pulse VA_TMS_ conditions. Another two-way ANOVA was used to compare the linearity of the voluntary moment and SIT relation between the SCI and non-impaired groups; this comparison was tested without excluding low linearity blocks. ANOVA assumptions were tested using Shapiro-Wilk normality tests and visually inspecting residuals. Tukey HSD multiple comparison tests were used for *post hoc* analyses. Finally, we reported the percent of trials that met the Todd et al. (2016) criteria (biceps MEP ≥ 50% Mmax and triceps MEP ≤ 20% Mmax) and an adjusted condition (MEP ratio ≥ 2.5) to account for the triceps being in a higher susceptibility state during VA_TMS_ trials. All data are presented as mean ± standard error of the mean unless stated otherwise. Statistical significance was set at the *p* < 0.05 level.

## Results

**FIGURE 3:**
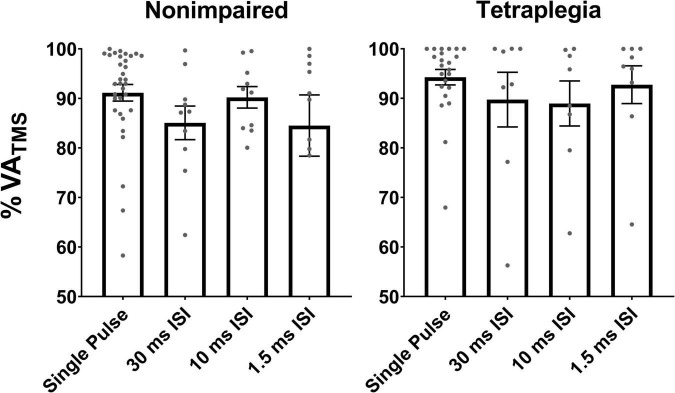
VA_TMS_ measures collected during the single pulse and paired pulse conditions in non-impaired and SCI participants. Gray points represent individual mean VA_TMS_ (per block). VA_TMS_ ranged from 56 to 99%. Error bars represent the standard error of the mean.

Across all participants with SCI, mean VA_TMS_ collected with single pulse TMS was 94.3 ± 7.7% and mean VA_PNS_ was 95.7 ± 7.1%. Mean VA_TMS_ was 92.7 ± 11.0% with the 1.5 ms condition, 88.9 ± 13.2% with the 10 ms ISI condition, and 89.7 ± 15.0% with the 30 ms ISI condition (Figure 3). Across all non-impaired participants, mean VA_TMS_ collected with single pulse TMS was 91.1 ± 5.3% and mean VA_PNS_ was 98.2 ± 3.3%. For paired pulse stimulation, mean VA_TMS_ was 84.5 ± 7.7% with the 1.5 ms ISI condition, 90.2 ± 7.9% with the 10 ms ISI condition, and 85.1 ± 7.9% with the 30 ms ISI condition (Figure 3). The linearity of the voluntary moment and SIT moment relation (*r*-value) was on average lower in the SCI group compared to the non-impaired group across stimulation pulses [*F*_(1_, _103)_ = 7.043, *p* = 0.0092] (Table 2). In the SCI group, 23 out of 49 VA_TMS_ blocks demonstrated a poor linear fit (*r* < 0.8) between the voluntary moment and SIT moment. Although data were not excluded in the SCI group analyses based on linearity, we calculated VA_TMS_ for each condition if data had been excluded for this reason, which is available in Supplementary Text in Supplementary material. Average biceps RMTs were 37.6 ± 10.8% MSO for the SCI group and 44.3 ± 14.1% MSO for the non-impaired group (see Supplementary Tables 8, 9 in Supplementary material for individual participant RMTs for each session). The datasets and Supplementary material for this study can be can be accessed online at https://osf.io/sdxj9/.

**TABLE 2:** Percent trials (between 50 and 100% MVC) meeting the Todd et al. criteria (biceps MEP ≥ 50% Mmax and triceps MEP ≤ 20% Mmax), MEP ratio > 2.5 (where biceps MEP is 2.5 larger than triceps MEP), and the average linearity of the voluntary moment and SIT moment.

Stimulation pulse	Todd et al. criteria (% met)	MEP ratio ≥ 2.5 (% met)	Mean linearity	Total # of trials
**Non-impaired**				
Single pulse	34.1	60.2	0.87	540
Paired 1.5 ms ISI	42.8	68.9	0.78	180
Paired 10 ms ISI	41.9	69.7	0.81	198
Paired 30 ms ISI	39.4	61.7	0.81	180
Mean	*39.5*	*65.1*	0.83*	–
**SCI**				
Single pulse	14.4	52.8	0.81	432
Paired 1.5 ms ISI	20.0	68.9	0.65	180
Paired 10 ms ISI	27.8	60.4	0.74	144
Paired 30 ms ISI	15.3	34.0	0.71	144
Mean	*19.4*	*54.0*	0.73*	–

*Indicate statistically different values (*p* < 0.05).

### Effect of independent variables on VA_TMS_

In the SCI group, the main effect of the stimulation pulse (1.5, 10, and 30 ms ISI compared to single pulse) on VA_TMS_ was not significant in the linear mixed-effects model. The main effect of the linearity of the voluntary moment and SIT moment relation on VA_TMS_ was significant in the linear mixed-effects model. For each 0.1 increase in linearity (for 0 < *r* < 0.99), VA_TMS_ was predicted to increase by 7.5% [*t* = 7.005, *p* < 0.001]. Further analyses revealed that the mean MEP ratio, RMT had no significant main effects on VA_TMS_ as well as no interaction effects with the stimulation pulse. In the non-impaired group, the main effect of stimulation pulse (1.5, 10, and 30 ms ISI compared to single pulse) on VA_TMS_ was not significant in the linear mixed-effects model. Further analyses revealed that the mean MEP ratio, RMT, and linearity had no significant main effects on VA_TMS_ as well as no interaction effects with the stimulation pulse.

### Effect of stimulation pulse on the biceps/triceps motor evoked potential ratio

**FIGURE 4:**
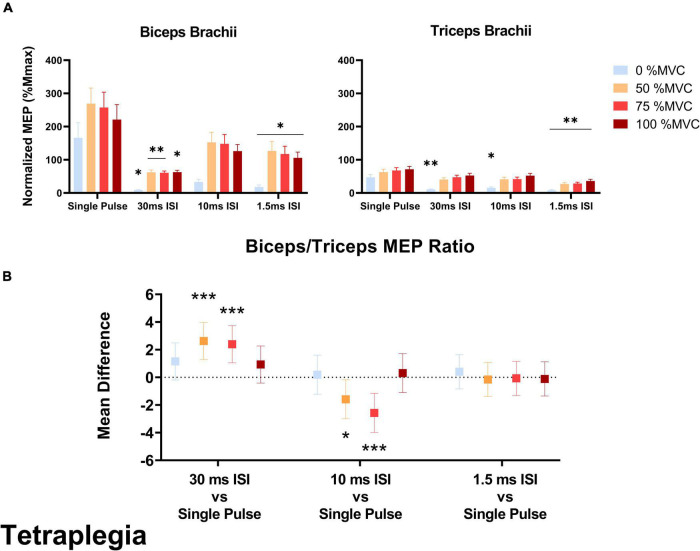
**(A)** Average biceps and triceps normalized MEPs (normalized to corresponding Mmax) in the SCI group. In the biceps, a significant decrease was observed for MEP amplitudes in 30 and 1.5 ms ISI conditions compared to single pulse. In the triceps, 30 and 10 ms ISI conditions led to lower MEPs but only at rest while 1.5 ms ISI led to lower MEPs across all effort levels. Error bars represent the standard error of the mean. Asterisks indicate statistical significance compared to the single pulse condition. **(B)** Biceps/Triceps MEP ratio mean difference relative to single pulse TMS in the SCI group. Errors bars show 95% confidence intervals. Asterisks indicate a significantly different mean MEP ratio ([*] = *p* < 0.05, [**] = *p* < 0.01, [***] = *p* < 0.001) relative to the mean MEP ratio with single pulse TMS.

**FIGURE 5:**
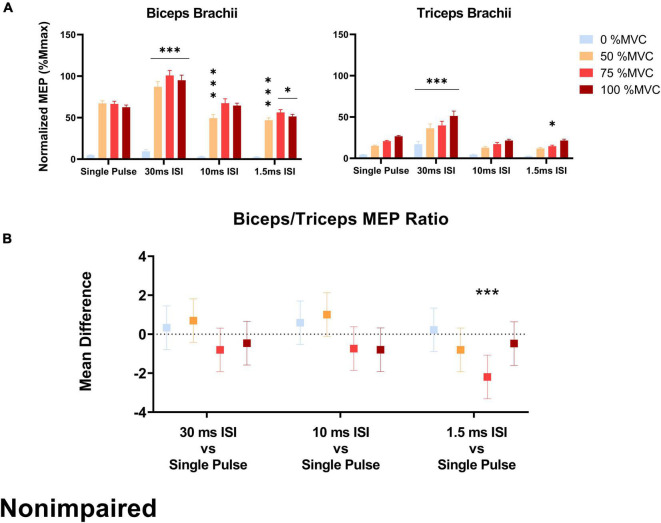
**(A)** Average biceps and triceps MEPs (normalized to corresponding Mmax) across stimulation conditions and effort levels in the non-impaired group. Biceps and triceps MEPs were increased during the 30 ms ISI condition while 10 and 1.5 ms ISI led to lower MEPs but not across all effort levels. Error bars represent the standard error of the mean. Asterisks indicate statistical significance compared to the single pulse condition. **(B)** MEP ratio mean difference relative to single pulse TMS across effort levels in the non-impaired group, only 1.5 ms ISI led to an increased MEP ratio at 75% MVC. Errors bars show 95% confidence intervals. Asterisks indicate a significantly different mean MEP ratio ([*] = *p* < 0.05, [***] = *p* < 0.001) relative to the mean MEP ratio with single pulse TMS.

In the SCI group, the biceps/triceps MEP ratio was increased in the 10 ms ISI condition relative to the baseline single pulse condition at 50% MVC (*t* = 2.205, *p* = 0.020) and 75% MVC (*t* = 3.571, *p* < 0.001; Figure 4). The MEP ratio was also decreased in the 30 ms ISI condition relative to the baseline single pulse condition at 50% MVC (*t* = 3.851, *p* < 0.001) and 75% MVC (*t* = 3.506, *p* < 0.001; Figure 4B). In the non-impaired group, the biceps/triceps MEP ratio was increased in the 1.5 ms (*t* = 3.849, *p* = 0.0001) condition relative to the baseline single pulse condition only at 75% MVC (Figure 5B).

### Effect of stimulation pulse on biceps motor evoked potentials

In the SCI group, 30 ms ISI [–158% Mmax (percent change to single pulse), *p* = 0.018] and 1.5 ms ISI [–148% Mmax, *p* = 0.016] decreased biceps MEPs collected at rest. At 50% MVC, 30 ms ISI [–207% Mmax, *p* = 0.0019] and 1.5 ms ISI [–142% Mmax, *p* = 0.020] decreased biceps MEPs compared to single pulse. At 75% MVC, 30 ms ISI [–197% Mmax, *p* = 0.003] and 1.5 ms ISI [–140% Mmax, *p* = 0.022] decreased biceps MEPs compared to single pulse. At 100% MVC, only 30 ms ISI decreased biceps MEPs [–158% Mmax, *p* = 0.017] compared to single pulse (Figure 4A).

In the non-impaired group, stimulation pulse had no effect on biceps MEPs collected at rest. At 50% MVC, 30 ms ISI increased biceps MEPs [+ 20.04% Mmax, *p* < 0.001] while 1.5 ms ISI [–20.2% Mmax, *p* < 0.001] and 10 ms ISI [–17.7% Mmax, *p* < 0.001] decreased biceps MEPs compared to single pulse. At 75% MVC, 30 ms ISI increased biceps MEPs [+ 34.3% Mmax, *p* < 0.001] compared to single pulse. At 100% MVC, 30 ms ISI increased biceps MEPs [+ 32.6% Mmax, *p* < 0.001] while 1.5 ms ISI decreased biceps MEPs 5 [–11.1% Mmax, *p* = 0.023] compared to single pulse (Figure 5A).

### Effect of stimulation pulse on triceps motor evoked potentials

In the SCI group, 30 ms ISI [–36.2% Mmax, *p* = 0.004], 10 ms ISI [–31.5% Mmax, *p* = 0.019], and 1.5 ms ISI [–38.9% Mmax, *p* < 0.001] decreased triceps MEPs collected at rest. At 50% MVC, only 1.5 ms ISI decreased triceps MEPs compared to single pulse [–35.7% Mmax, *p* = 0.002]. At 75% MVC only 1.5 ms ISI decreased triceps MEPs compared to single pulse [–39.1% Mmax, *p* < 0.001]. At 100% MVC, only 1.5 ms ISI decreased triceps MEPs [–35.2% Mmax, *p* = 0.003] compared to single pulse (Figure 4A).

In the non-impaired group, only 30 ms ISI increased triceps MEPs collected at rest [+ 12.7% Mmax, *p* < 0.001]. At 50% MVC, 30 ms ISI increased triceps MEPs [+ 21.2% Mmax, *p* < 0.001]. At 75% MVC, 30 ms ISI increased triceps MEPs [+ 19.1% Mmax, *p* < 0.001] while 1.5 ms ISI decreased triceps MEPs 5 [–6.09% Mmax, *p* = 0.029] compared to single pulse. At 100% MVC, only 30 ms ISI increased triceps MEPs [+ 24.6% Mmax, *p* < 0.001] compared to single pulse (Figure 5A).

### Evaluation of biceps/triceps motor evoked potential ratio

In both the SCI and non-impaired groups, the 10 and 1.5 ms ISI pulses had a higher number of trials that met the guideline criteria presented by Todd et al. (2016) (biceps MEP ≥ 50% Mmax and triceps MEP ≤ 20% Mmax) and our adjusted condition of corresponding to a MEP ratio greater than 2.5 (Table 2). Across both groups and all stimulation pulses, the “MEP ratio > 2.5” condition was met more often than the Todd et al. (2016) criteria.

## Discussion

We used paired pulse TMS techniques as an approach to test for a relationship between the biceps/triceps MEP ratio and biceps VA_TMS_ in muscle affected by SCI. In evaluating individuals with C5 and C6 tetraplegia, we hypothesized that paired pulse TMS with ISI that can facilitate biceps MEPs would increase the biceps/triceps MEP ratio across all effort levels relative to single pulse TMS; this hypothesis was not supported. We also hypothesized that paired pulse TMS with ISI that can inhibit biceps MEPs would decrease the biceps/triceps MEP ratio across all effort levels relative to single pulse TMS in the SCI participants; this hypothesis was not supported. Further, we hypothesized that the biceps/triceps MEP ratio would affect VA_TMS_ in individuals with tetraplegia; this hypothesis was also not supported. Thus, the biceps/triceps MEP ratio may not contribute to greater excitation of the biceps and larger SIT elbow moments during voluntary contraction. In the SCI group, VA_TMS_ was found to be sensitive to the linearity of the SIT moment and voluntary moment relation. Linearity was lower in the SCI group compared to the non-impaired group, which poses a methodological limitation in the estimation of VA_TMS_ after SCI. Further research is needed to determine whether VA_TMS_ is a viable assessment of neuromuscular function after SCI. For future work probing facilitation and inhibition at high effort levels of muscle contraction, we recommend studies focused on optimization of paired pulse TMS parameters specific to the effort level and muscle.

While the target/antagonist MEP ratio may be an indication of cortical stimulation focality when muscles are at rest or at low levels of activation, this relationship may not hold at higher levels of effort during stimulation. Increasing the level of muscle contraction to about 20% MVC leads to a greater proportion of spinal motoneurons activated by TMS, which increases the sensitivity of spinal motorneurons to changes in corticospinal excitability (Di Lazzaro et al., 1998; Martin et al., 2006). When the biceps are highly activated (75–100% MVC), the triceps are not at rest, but experiencing low levels of activation (i.e., increased EMG activity compared to baseline as seen in Figure 2). The biceps during these high-level efforts, being at a near tetanic state, are at suboptimal capacity to elicit a larger MEP response. Indeed, prior studies demonstrate saturation of MEPs during voluntary contraction in non-impaired muscle (Valls-Sole et al., 1994; Van den Bos et al., 2018). Conversely, the triceps, being in a lower activation state, not only have an increased capacity to respond (i.e., are further from a tetanic state) but are in a state of higher excitability compared to rest (e.g., active motor thresholds are lower than resting motor thresholds) (Todd et al., 2006; Ah Sen et al., 2017; Kesar et al., 2019). Consequently, the Todd et al. (2016) criteria (i.e., biceps MEP ≥ 50% Mmax and triceps MEP ≤ 20% Mmax) were seldom met (Table 2, non-impaired: 39.9% of trials, SCI: 19.4% trials). We proposed an adjusted condition (MEP ratio ≥ 2.5), reflective of the relative responsiveness between both muscles to account for the triceps being in a higher excitability state during biceps VA_TMS_ trials. Compared to the Todd et al. (2016) criteria, this adjusted condition may better reflect the relative contribution of the biceps and the triceps to the SIT at high effort levels. This condition was met more often in both groups (SCI: 54.0% trials, non-impaired: 65.1% of trials), especially in 1.5 ms ISI trials where both groups met the condition more than two-thirds of the time (see Table 2). However, neither paired pulse stimulation nor the MEP ratio affected the estimation of VA_TMS_. Therefore, while the MEP ratio may be physiologically relevant at rest and at efforts up to about 20% MVC, its utility and importance are reduced at higher effort levels where the target and antagonist muscles are asymmetrically activated and responsive to TMS.

Overall the results in the non-impaired biceps were largely consistent with previous reports where MEP responses were inhibited with short ISI (1.5 ms) and facilitated at a longer ISI of 30 ms (Figure 5A) (Kujirai et al., 1993; Hunter et al., 2016). Previous research suggests that low amounts of voluntary activation (∼20% MVC) of the target muscle decrease SICI (i.e., less inhibition compared to rest) and ICF (Ridding et al., 1995). Our protocol involved high effort levels (50–100% MVC) where we observed both SICI and ICF. Unexpectedly, 10 ms ISI resulted in biceps MEP inhibition although such an ISI can elicit MEP facilitation (Ziemann, 1999; Ilić et al., 2002). However, MEP ratio modulation did not occur across all effort levels in the non-impaired group due to inconsistent facilitation and inhibition effects across effort levels, and MEP facilitation that occurred simultaneously in the biceps and the triceps. In the non-impaired group, 30 ms ISI increased both biceps and triceps MEPs across all effort levels compared to single pulse. Since MEP facilitation occurred simultaneously in the biceps and triceps, the biceps/triceps MEP ratio remained unchanged relative to single pulse. The only condition in which the MEP ratio was modulated in the non-impaired group was at 75% MVC with an ISI of 1.5 ms that induced triceps inhibition. Unexpectedly, 10 ms ISI resulted in biceps MEP inhibition although such an ISI can elicit MEP facilitation (Ziemann, 1999; Ilić et al., 2002).

MEP ratio modulation also did not occur across all effort levels in the SCI group due to inconsistent facilitation and inhibition across effort levels. Injury-induced reorganization of the corticospinal pathways after SCI affects inhibitory and facilitatory circuits and can lead to unpredictable paired pulse TMS outcomes in resting muscle (Oudega et al., 2012; Nardone et al., 2013, 2015; Vastano and Perez, 2020). Unexpectedly in the SCI group, the biceps/triceps MEP ratio was decreased in the 30 ms ISI condition compared to the single pulse protocol at 50 and 75% MVC; this occurred mostly via biceps MEP inhibition (–180% Mmax across all effort levels). Further, the MEP ratio was increased in the 10 ms ISI condition, which is the only condition that did not elicit significant biceps MEP inhibition compared to single pulse. Following SCI, death of motoneurons and changes in the properties of remaining motoneurons will affect their behavior during voluntary efforts (D’Amico et al., 2014; Grumbles and Thomas, 2017). Specifically, the excitability of motoneurons increases during voluntary contractions to a lesser extent than in non-impaired (Vastano and Perez, 2020). SICI can be elicited in individuals with SCI during voluntary efforts but MEP inhibition is reduced compared to non-impaired controls (Roy et al., 2011). In our study, SICI occurred at 1.5 ms ISI in the biceps but also in the triceps at high effort levels leading to an unchanged MEP ratio. As previously described, during biceps MVC, the biceps is closer to its maximal firing rate and thus less sensitive to stimulation whereas the triceps is in a more receptive state. In such context, SICI appears to decrease triceps MEPs preferentially. This asymmetric response may also be influenced by increased reticulospinal inputs to the biceps and decreased corticospinal inputs to the triceps following SCI (Sangari and Perez, 2019, 2020).

In the SCI group, we observed abnormal MEP inhibition in paired pulse trials at 30 ms ISI that elicited facilitation in non-impaired participants. While there is evidence to suggest that the excitability of inhibitory circuits mediated by the activity of GABA-A receptors is reduced after SCI as a compensatory mechanism (Norton et al., 2008; Boulenguez et al., 2010; Roy et al., 2011), the effects of ICF neurophysiology following SCI have not been well documented. One possible interpretation is that 30 ms ISI caused long interval intracortical inhibition (LICI) in our SCI cohort instead of ICF. LICI is mediated by the activity of GABA-B receptors (McDonnell et al., 2006) and is typically elicited at ISIs ≥ 50 ms and with suprathreshold conditioning pulse (Nakamura et al., 1997). However, animal studies have shown that the expression of GABA-B receptor is altered following SCI (Romaus-Sanjurjo et al., 2018). Further, active LICI (during voluntary contractions) was found to be increased in participants with SCI compared to non-impaired controls in FDI muscles (Barry et al., 2013). Mechanisms below the cortical level may be involved as well. After SCI, the presence of axonal dysfunction of the descending corticospinal tract and peripheral motor axon dysfunction indicates that both central and peripheral pathways can contribute to aberrant modulation of MEPs (Lin et al., 2007; Van De Meent et al., 2010).

Linearity of the SIT moment and voluntary moment relation was lower in the SCI group compared to the non-impaired group (0.73 vs 0.83, see Table 2), which reveals a methodological issue that limits the interpretation of VA_TMS_ in our SCI cohort. Similar to data reported by Todd et al. (2003) (VA_TMS_ = 93.6 ± 5.6%), single pulse VA_TMS_ was in the 90–95% range on average, in both groups (non-impaired: 91.1%, SCI: 94.3%). Paired pulse stimulation did not affect the estimation of VA_TMS_ compared to single pulse (Figure 3). Lower linearity (*r* < 0.8), on the other hand, decreased estimation of VA_TMS_ in the SCI group. Todd et al. (2016) recommend a linear relationship (*r* ≥ 0.9) for effort levels of 50–100% MVC to extrapolate the resting twitch moment and properly calculate VA_TMS_. Thus, interpretation of VA_TMS_ is especially difficult in a context that affects linearity, including the biceps in individuals with C5 and C6 tetraplegia.

Motor unit recruitment is altered after SCI and should be considered in future work investigating MEP modulation and VA_TMS_. Motor units are recruited with voluntary effort and with TMS according to Henneman’s size principle (Henneman, 1958; Bawa and Lemon, 1993). In non-impaired muscle during a MVC, force is mainly generated by gradation of motor unit firing rate (Fuglevand et al., 1999). However, in hand muscle affected by cervical SCI, motor unit recruitment becomes more important for force production: recruitment occurs over an expanded range of forces (up to 85% MVC in hand muscles), and after recruitment, motor units show little or no increase in firing rate (Zijdewind and Thomas, 2003). Further, relative to non-impaired muscle, TMS may activate different descending pathways after SCI, and afferent feedback during muscle contraction may differentially alter spinal motorneuron excitability and motor unit recruitment (Zijdewind et al., 2012). A greater understanding of motor unit recruitment with VA_TMS_ in more proximal muscles such as the biceps in tetraplegia is needed.

## Limitations

There are limitations in this preliminary study. The sample size is small and there was a wide range of biceps strength among the SCI participants. Some participants presented more overall remaining biceps strength as indicated by greater maximum elbow flexor moments. Yet, VA_TMS_ measures across SCI and non-impaired participants were in the same range (see Figure 3) which suggests that VA_TMS_ did not detect differences in motor impairments, or that the individuals with SCI did not have denervated muscle that could be accessed with neurostimulation. While the number of motor units and their maximal firing rates may decrease in the biceps after SCI resulting in lower force-generating capacity (Thomas et al., 2014), the relative amount of innervated muscle fibers able to receive descending voluntary neural drive may be unchanged. However, in a context where corticomotor transmission and excitability are affected such as in tetraplegia, TMS capacity to elicit moment twitches is reduced, resulting in poor linearity and potential overestimation of VA_TMS_; thus, VA_TMS_ is difficult to interpret. Another reason why interpretation of VA_TMS_ is difficult is that although it may be expected for VA_TMS_ to be greater relative to VA_PNS_ in non-impaired muscle (since VA_TMS_ represents deficits in cortical activation, and VA_PNS_ represents cortical, corticospinal, and peripheral deficits) VA_TMS_ has been reported to be lower relative to VA_PNS_ in unfatigued and fatigued biceps (Todd et al., 2003; Cadigan et al., 2017). Similarly, biceps VA_TMS_ was lower compared to VA_PNS_ in both groups (SCI and non-impaired) in the current study. Estimation of VA_TMS_ remains a methodological challenge (Todd et al., 2016).

Another limitation is that we did not optimize the paired pulse TMS parameters (i.e., ISIs, conditioning, and test stimulus intensities) for each level of muscle activation, nor did we account for the potential differences in optimal parameters between SCI and non-impaired participants. Future work including systematic investigation of optimal stimulus parameters that selectively modulate the biceps MEP relative to the triceps MEP at each of level of activation may induce a wider range of biceps/triceps MEP ratios for which to assess the relationship with VA_TMS_. With the stimulus parameters we used, which were based on previous investigations of resting muscle, we did induce facilitation and inhibition, but facilitation and inhibition were inconsistent across levels of muscle activation. Another limitation is that we did not exclude data based on poor linearity in the SCI group. Rather, we report the effect of linearity on VA_TMS_ in muscle affected by SCI and acknowledge that the inability to satisfy an important underlying assumption of the technique (i.e., a linear relationship between the voluntary moment and SIT) is a major challenge that precludes a meaningful interpretation of VA_TMS_. In line with this limitation, we did not compare VA_TMS_ between groups. Another potential limitation is that we designed our experiment to have more rest, more often (90 s rest between each trial), between voluntary effort trials compared to previous work (Todd et al., 2003; Cadigan et al., 2017), fatigue and attention may have affected our results, especially in SCI participants as previously discussed. Since our experiments were designed to measure VA_TMS_, single pulse MEPs (test only) and paired pulse MEPs (conditioned and test) were collected in separate blocks. This is important since unlike previous work (Hanajima et al., 1998; Vahabzadeh-Hagh, 2014), comparison was performed at the block level and not on a trial-to-trial basis. Thus, MEP data were averaged per stimulation condition before comparison. Participants were not age-matched; however, no direct comparisons were made between groups when analyzing MEPs and VA_TMS_ and previous work suggests that age does not influence voluntary activation and paired pulse TMS outcomes (Klein et al., 2001; Van den Bos et al., 2018). Finally, we did not measure spasticity in the biceps and triceps in SCI particicpants, although no visible signs of spasticity were present.

## Conclusion

As a novel contribution, we assessed VA_TMS_ using paired pulse TMS in individuals with tetraplegia, although methodological issues remain that need further investigation. Paired pulse TMS did not modulate the biceps/triceps MEP ratio across the full range of voluntary efforts participants with tetraplegia, and did not affect the estimation of VA_TMS_. Thus, a focus on increasing the biceps/triceps MEP ratio via paired pulse stimulation may not improve the estimation of VA_TMS_ after SCI. In participants with tetraplegia, paired pulse TMS revealed different patterns of intracortical inhibition relative to non-impaired participants that may be due to injury-induced corticospinal reorganization and alterations in the activity of GABA-B receptors following SCI. More comprehensive paired pulse TMS experiments across levels of activation are needed to further our understanding of neuroplastic changes and functional reorganization after SCI. For future work probing facilitation and inhibition at high effort levels of muscle contraction, we recommend studies focused on optimization of paired pulse TMS parameters specific to the effort level and muscle. Finally, VA_TMS_ was sensitive to the linearity of the voluntary moment and SIT moment relation in participants with tetraplegia; linearity was lower compared to non-impaired, which constitutes a fundamental challenge in the estimation of VA_TMS_ after SCI. Further research is needed to determine whether VA_TMS_ is a viable assessment of neuromuscular function in individuals with tetraplegia.

## Data availability statement

The datasets presented in this study can be found in online repositories. The names of the repository/repositories and accession number(s) can be found in the article/Supplementary material.

## Ethics statement

The studies involving human participants were reviewed and approved by Virginia Commonwealth University Institutional Review Board (HM20010929). The patients/participants provided their written informed consent to participate in this study.

## Author contributions

TR contributed to software, methodology, validation, formal analysis, investigation, data collection, data curation, writing (original draft), writing (review and editing), and visualization. BT contributed to validation, investigation, writing (original draft), and writing (review and editing). CP contributed to conceptualization, methodology, resources, and writing (review and editing), supervision, project administration, and funding acquisition. All authors contributed to the study conception and design.
