# Supplementary material for: Paired pulse transcranial magnetic stimulation in the assessment of biceps voluntary activation in individuals with tetraplegia
Source: Front Hum Neurosci. 2022 Nov 3;16:976014. doi: 10.3389/fnhum.2022.976014 (PMC9669314; doi:10.3389/fnhum.2022.976014)
Supplement: Supplementary file 1 [file Data_Sheet_1.docx]

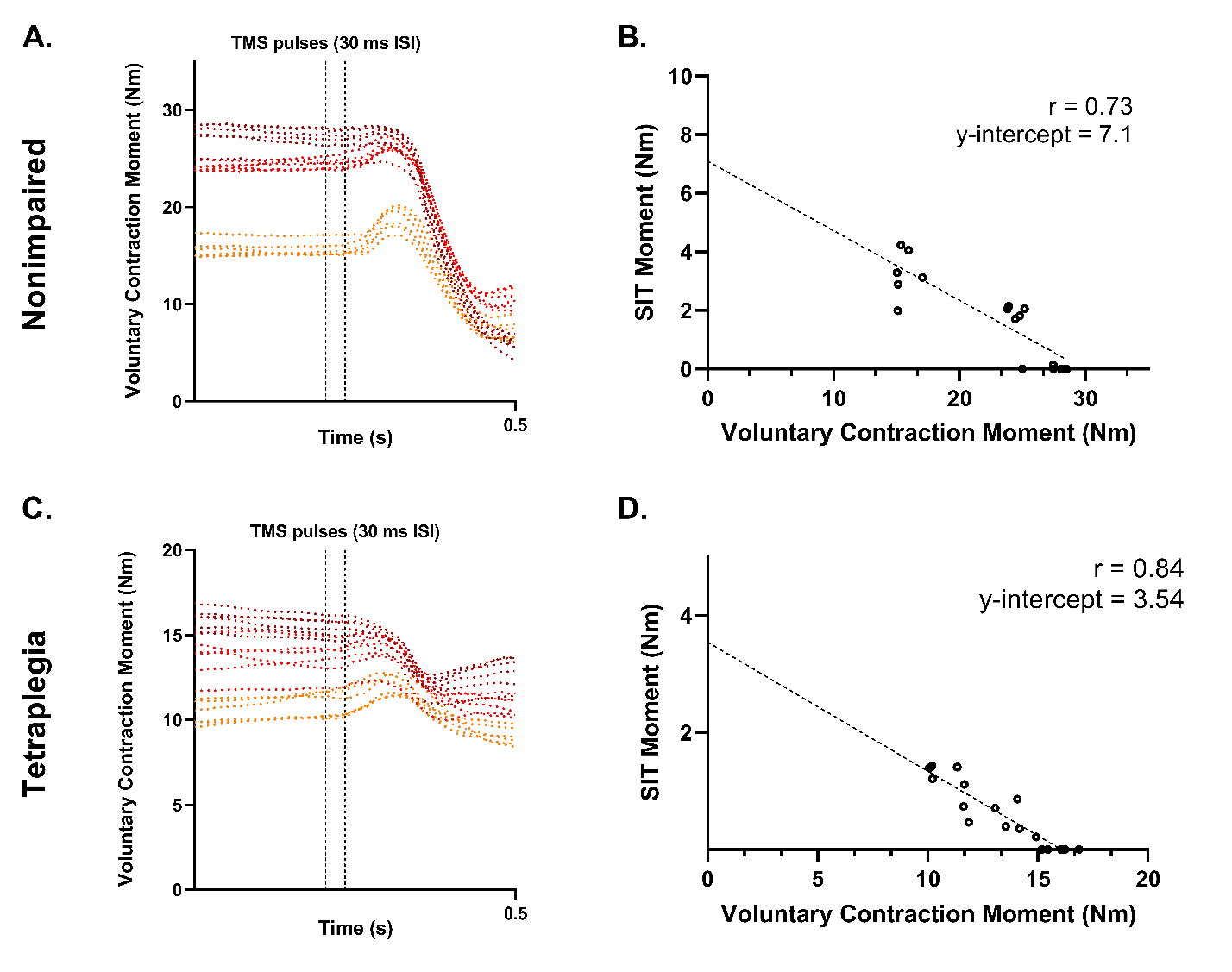


**S1 Figure.** VA_TMS_ with paired pulse TMS at 30 ms ISI example data. **A.** Moment traces during a VA_TMS_ block in a representative nonimpaired participant. **B.** Linear regression between SIT and voluntary contraction moment from the same VA_TMS_ block. **C.** Moment traces during a VA_TMS_ block in a representative participant with tetraplegia. **D.** Linear regression between SIT and voluntary contraction moment obtained from the same VA_TMS_ block. Orange, red, and dark red dotted lines represent 50, 75, and 100% MVC trials, respectively.





**S2 Figure.** VA_TMS_ with paired pulse TMS at 10 ms ISI example data. **A.** Moment traces during a VA_TMS_ block in a representative nonimpaired participant. **B.** Linear regression between SIT and voluntary contraction moment from the same VA_TMS_ block. **C.** Moment traces during a VA_TMS_ block in a representative participant with tetraplegia. **D.** Linear regression between SIT and voluntary contraction moment obtained from the same VA_TMS_ block. Orange, red, and dark red dotted lines represent 50, 75, and 100% MVC trials, respectively.





**S3 Figure.** VA_TMS_ with paired pulse TMS at 1.5 ms ISI example data. **A.** Moment traces during a VA_TMS_ block in a representative nonimpaired participant. **B.** Linear regression between SIT and voluntary contraction moment from the same VA_TMS_ block. **C.** Moment traces during a VA_TMS_ block in a representative participant with tetraplegia. **D.** Linear regression between SIT and voluntary contraction moment obtained from the same VA_TMS_ block. Orange, red, and dark red dotted lines represent 50, 75, and 100% MVC trials, respectively.





**S4 Figure.** Example of a single pulse VA_TMS_ block with low linearity collected from a participant with tetraplegia. **A.** Moment traces where orange, red, and dark red dotted lines represent 50, 75, and 100% MVC trials, respectively. **B.** Linear regression between SIT and voluntary contraction moment from the same VAtms block.

**S5 Text.**

If data were excluded in SCI participants per poor linearity (r < 0.8), the mean VA_TMS_ collected with single pulse TMS was 95.4 ± 7.74 %. For the paired pulse paradigms, if data were excluded in SCI participants per poor linearity (r < 0.8), mean VA_TMS_ was 98.1 ± 2.2 % with the 1.5 ms condition, 93.8 ± 9.7 % with the 10 ms ISI condition, and 99.7 ± 0.4 % with the 30 ms ISI condition.

**S6 Table.** Biceps and Triceps peak-to-peak Mmax values and corresponding stimulator intensities for each session in the Nonimpaired group.

| NI | Biceps Mmax PP (uV) | | | Biceps Stimulation Intensity (mA) | | | Triceps Mmax PP (uV) | | | Triceps Stimulation Intensity (mA) | | |
| --- | --- | --- | --- | --- | --- | --- | --- | --- | --- | --- | --- | --- |
|  | Sess. 1 | Sess. 2 | Sess. 3 | Sess. 1 | Sess. 2 | Sess. 3 | Sess. 1 | Sess. 2 | Sess. 3 | Sess. 1 | Sess. 2 | Sess. 3 |
| NI01 | 4992 | 4571 | 5874.2 | 110 | 70 | 80 | 1397 | 1129 | 2089.5 | 100 | 100 | 100 |
| NI02 | 1269.8 | 2327.3 | 1710.6 | 60 | 70 | 70 | 1018 | 929.9 | 808.1 | 90 | 90 | 100 |
| NI03 | 1491.8 | 1406.5 | 1666.7 | 90 | 80 | 80 | 802.7 | 1054.1 | 1149.5 | 90 | 80 | 90 |
| NI04 | 1878.7 | 2095.5 | 3812.9 | 80 | 110 | 90 | 1178 | 571 | 1050.1 | 130 | 110 | 90 |
| NI05 | 992 | 5246.4 | 2049.4 | 100 | 130 | 130 | 844 | 567 | 614.9 | 110 | 80 | 130 |
| NI06 | 4797.1 | 4288.6 | 5362.9 | 90 | 110 | 110 | 1085 | 508.3 | 1180.7 | 70 | 120 | 120 |
| NI07 | 4019.2 | 2578.7 | 2472.9 | 70 | 80 | 80 | 1211.3 | 736.8 | 1054.2 | 90 | 80 | 80 |
| NI08 | 10670 | 4017 | 6939 | 90 | 80 | 80 | 1542.1 | 1805.6 | 3410.7 | 60 | 80 | 110 |
| NI09 | 1193.9 | 2413.7 | 3864.3 | 90 | 100 | 80 | 1379.8 | 1111.8 | 1424.8 | 120 | 100 | 100 |
| NI10 | 4748.9 | 8706.3 | 7903.5 | 90 | 70 | 80 | 3417.6 | 1974 | 1546.1 | 100 | 100 | 80 |

**S7 Table.** Biceps and Triceps peak-to-peak Mmax values and corresponding stimulator intensities for each session in the Tetraplegia group.

| SCI | Biceps Mmax PP (uV) | | | Biceps Stimulation Intensity (mA) | | | Triceps Mmax PP (uV) | | | Triceps Stimulation Intensity (mA) | | |
| --- | --- | --- | --- | --- | --- | --- | --- | --- | --- | --- | --- | --- |
|  | Sess. 1 | Sess. 2 | Sess. 3 | Sess. 1 | Sess. 2 | Sess. 3 | Sess. 1 | Sess. 2 | Sess. 3 | Sess. 1 | Sess. 2 | Sess. 3 |
| SCI01 | 1026.88 | 1149.95 | 410.15 | 140 | 130 | 120 | 226.6 | 1122.6 | 411.6 | 140 | 130 | 120 |
| SCI02 | 246.09 | 212.16 | 1016.98 | 140 | 120 | 130 | 433.5 | 99.5 | 136.7 | 140 | 120 | 120 |
| SCI03 | 214.37 | 419.13 | 189.24 | 160 | 150 | 160 | 147.1 | 147.5 | 224 | 120 | 150 | 150 |
| SCI04 | 1561.17 | 1368.82 | 2662.58 | 110 | 130 | 120 | 1307.8 | 1858.3 | 1905.9 | 100 | 130 | 120 |
| SCI05 | 969.41 | 280.57 | 386.38 | 130 | 150 | 120 | 422.7 | 347 | 401.6 | 130 | 150 | 120 |
| SCI06 | 2455.8 | 1315.8 | N/A | 130 | 100 | N/A | 166.9 | 228.3 | N/A | 130 | 70 | N/A |
| SCI07 | 9530.9 | 10232 | N/A | 90 | 80 | N/A | 4559.8 | 2092.2 | N/A | 100 | 80 | N/A |
| SCI08 | 4219.9 | 1558.4 | N/A | 100 | 120 | N/A | 954.7 | 1596.7 | N/A | 120 | 120 | N/A |
| SCI09 | 683.5 | 595 | 707.5 | 140 | 160 | 150 | 278.1 | 291 | 134.5 | 140 | 160 | 150 |
| SCI10 | 620.4 | 435.44 | N/A | 120 | 120 | N/A | 1485.5 | 1925.9 | N/A | 130 | 120 | N/A |

**S8 Table.** Biceps Resting Motor Thresholds (RMTs) in the Nonimpaired group.

| NI | Biceps RMT (%MSO) | | |
| --- | --- | --- | --- |
|  | Sess. 1 | Sess. 2 | Sess. 3 |
| NI01 | 50 | 51 | 50 |
| NI02 | 60 | 35 | 37 |
| NI03 | 21 | 21 | 20 |
| NI04 | 53 | 53 | 46 |
| NI05 | 71 | 74 | 74 |
| NI06 | 48 | 56 | 50 |
| NI07 | 40 | 47 | 47 |
| NI08 | 37 | 47 | 39 |
| NI09 | 37 | 33 | 34 |
| NI10 | 34 | 32 | 32 |

**S9 Table.** Biceps Resting Motor Thresholds (RMTs) in the Tetraplegia group.

| SCI | Biceps RMT (%MSO) | | |
| --- | --- | --- | --- |
|  | Sess. 1 | Sess. 2 | Sess. 3 |
| SCI01 | 63 | 52 | 52 |
| SCI02 | 34 | 32 | 28 |
| SCI03 | 37 | 39 | 38 |
| SCI04 | 43 | 42 | 36 |
| SCI05 | 49 | 54 | 52 |
| SCI06 | 28 | 30 | N/A |
| SCI07 | 32 | 27 | N/A |
| SCI08 | 26 | 32 | N/A |
| SCI09 | 26 | 26 | 26 |
| SCI10 | 26 | 27 | N/A |
